# 以泽布替尼为基础的桥接方案在CAR-T细胞治疗复发难治弥漫大B细胞淋巴瘤中的临床安全性及疗效分析

**DOI:** 10.3760/cma.j.issn.0253-2727.2023.10.004

**Published:** 2023-10

**Authors:** 燕 陆, 辉 刘, 世光 叶, 莉莉 周, 休 骆, 秀勇 党, 相贵 袁, 文斌 钱, 爱斌 梁, 萍 李

**Affiliations:** 1 同济大学附属同济医院血液科，上海 200065 Department of Hematology, Tongji Hospital, Tongji University School of Medicine, Shanghai 200065, China; 2 浙江大学附属第二医院血液科，杭州 310009 Department of Hematology, the Second Affiliated Hospital, College of Medicine, Zhejiang University, Hangzhou 310009, China

**Keywords:** 嵌合抗原受体T细胞, 泽布替尼, 桥接治疗, 复发/难治弥漫大B细胞淋巴瘤, Chimeric antigen receptor T-cells, Zanubrutinib, Bridging treatment, Relapsed or refractory diffuse large B-cell lymphoma

## Abstract

**目的:**

探讨以BTK抑制剂泽布替尼为基础的联合方案桥接CD19嵌合抗原受体T细胞（CAR-T细胞）治疗复发/难治弥漫大B细胞淋巴瘤（r/r DLBCL）的有效性和安全性。

**方法:**

回顾性分析同济大学附属同济医院血液科和浙江大学附属第二医院血液科在2020年6月至2023年6月期间收治的21例以泽布替尼为基础的方案桥接CD19 CAR-T细胞治疗伴有高危因素的r/r DLBCL患者，观察疗效及安全性。

**结果:**

研究共纳入21例患者，中位年龄为57（38～76）岁，14例（66.7％）美国东部肿瘤协作组体能状况评分（ECOG评分）≥2分，14例（66.7％）为双表达DLBCL，18例（85.7％）国际预后指数（IPI）评分≥3分，3例（14.3％）IPI评分2分的患者均存在结外受累，7例（33.3％）伴有TP53突变。中位随访24.8（95％*CI* 17.0～31.6）个月，客观缓解率（ORR）为81.0％，11例（52.4％）患者获完全缓解（CR）；中位无进展生存（PFS）时间为12.8个月，中位总生存（OS）时间未达到，1年PFS率为52.4％（95％*CI* 29.8％～74.3％），1年OS率为80.1％（95％*CI* 58.1％～94.6％）。18例（85.7％）患者发生了细胞因子释放综合征（CRS），均为1～2级；2例（9.6％）发生1级免疫效应细胞相关中枢神经毒性综合征（ICANS）。

**结论:**

以泽布替尼为基础的联合治疗作为CAR-T桥接方案治疗r/r DLBCL临床有效性高且安全性良好。

## Abstract (en)

**Objective:**

To further elucidate the clinical efficacy and safety of a combination regimen based on the BTK inhibitor zebutanil bridging CD19 Chimeric antigen receptor T cells（CAR-T cells）in the treatment of relapsed/refractory diffuse large B-cell lymphoma（r/r DLBCL）.

**Methods:**

Twenty-one patients with high-risk r/r DLBCL were treated with a zanubrutinib-based regimen bridging CAR-T between June 2020 and June 2023 at the Department of Hematology, Tongji Hospital, Tongji University and the Second Affiliated Hospital of Zhejiang University, and the efficacy and safety were retrospectively analyzed.

**Results:**

All 21 patients were enrolled, and the median age was 57 years（range: 38−76）. Fourteen patients（66.7％）had an eastern cooperative oncology group performance status score（ECOG score）of ≥2. Eighteen patients（85.7％）had an international prognostic index（IPI）score of ≥3. Three patients（14.3％）had an IPI score of 2 but had extranodal infiltration. Fourteen patients（66.7％）had double-expression of DLBCL and seven（33.3％）had TP53 mutations. With a median follow-up of 24.8（95％ *CI* 17.0−31.6）months, the objective response rate was 81.0％, and 11 patients（52.4％）achieved complete remission. The median progression-free survival（PFS）was 12.8 months, and the median overall survival（OS）was not reached. The 1-year PFS rate was 52.4％（95％ *CI* 29.8％−74.3％）, and the 1-year OS rate was 80.1％（95％*CI* 58.1％−94.6％）. Moreover, 18 patients（85.7％）had grade 1−2 cytokine-release syndrome, and two patients（9.5％）had grade 1 immune effector cell-associated neurotoxicity syndrome.

**Conclusion:**

Zanubrutinib-based combination bridging regimen of CAR-T therapy for r/r DLBCL has high efficacy and demonstrated a good safety profile.

弥漫大B细胞淋巴瘤（Diffuse large B cell lymphoma，DLBCL）是最常见的成人淋巴细胞肿瘤，具有侵袭性高和异质性大的临床特点。尽管50％～70％患者可通过标准一线治疗获得治愈，但仍有40％左右的患者对一线治疗难治或在缓解后疾病复发，预后非常差，中位总生存（Overall survival，OS）时间仅6个月，2年的OS率约20％[1]–[2]。近年来嵌合抗原受体T（Chimeric antigen receptor T，CAR-T）细胞治疗成为复发难治（Relapsed/refractory，r/r）DLBCL最有效的挽救治疗手段，总反应率达60％～70％，完全缓解（CR）率达40％～50％[3]。ZUMA-12临床研究的长期随访数据表明，约三分之一的经≥2线治疗的r/r DLBCL患者通过CAR-T治疗获得5年以上的持续缓解，即获得临床治愈的可能[4]。尽管r/r DLBCL的CAR-T治疗与传统治疗相比展现出显著的疗效，然而，中位无进展生存（Progression-free survival，PFS）时间仅6～7个月，大多数患者仍对CAR-T细胞耐药[3]。

目前国内外已有四款治疗r/r DLBCL的CAR-T细胞产品上市。CAR-T细胞的制备时间通常需要3～6周，约40％的r/r DLBCL患者由于疾病进展迅速，制备时间已超过治疗窗口[5]。有临床研究数据表明约7％的患者在CAR-T细胞制备期间出现死亡[6]。提示在单采后和预处理化疗前进行桥接治疗控制患者病情、阻止疾病的快速进展具有重要意义。另一方面，细胞输注前的肿瘤负荷大小是目前公认的CAR-T疗效的影响因素，同时也是影响细胞因子释放综合征（Cytokine release syndrome，CRS）严重程度的重要因素[7]。因此，也可以尝试通过桥接治疗来降低患者在细胞输注前的肿瘤负荷以提高CAR-T的疗效和安全性。

目前常用的桥接治疗方案包括化疗、小分子靶向药物单药或联合治疗、免疫治疗和放疗等。多项r/r套细胞淋巴瘤（Mantle cell lymphoma，MCL）和慢性淋巴细胞白血病（Chronic lymphocytic leukemia，CLL）的CAR-T临床研究数据表明在细胞输注前使用布鲁顿酪氨酸激酶抑制剂（Bruton tyrosine kinase inhibitors，BTKi）伊布替尼治疗可提高CAR-T细胞在体内的扩增，并可降低CRS严重程度[8]–[9]。泽布替尼是我国自主研发的具有更高选择性的BTKi，与BTK活性位点的半胱氨酸残基形成共价键以抑制BTK活性[10]。目前尚无以BTKi为基础的联合治疗作为CD19 CAR-T治疗r/r DLBCL桥接方案的研究报道。本研究我们分析了以泽布替尼为基础的联合治疗作为CD19 CAR-T治疗r/r DLBCL桥接方案的有效性和安全性。

## 病例与方法

一、病例

本回顾性研究纳入了2020年6月至2023年6月在同济大学附属同济医院血液科和浙江大学附属第二医院血液科行CD19 CAR-T治疗的伴有高危因素的r/r DLBCL患者21例。高危因素包括：结外受累、Ⅲ/Ⅳ期、乳酸脱氢酶升高（>250 U/L）、美国东部肿瘤协作组体能状况评分（Eastern Cooperative Oncology Group，ECOG评分）≥2分。所有患者均为非生发中心（Non-germinal center B-cell-like lymphoma，non-GCB）亚型。所有患者经病理组织学及免疫组织化学检查明确诊断为DLBCL。本研究获得同济大学附属同济医院伦理委员会和浙江大学附属第二医院伦理委员会批准。所有患者均签署了知情同意书。

二、治疗方法

17例患者的桥接方案为泽布替尼联合化疗±利妥昔单抗：利妥昔单抗375 mg/m^2^，d 0；泽布替尼160 mg每日2次，d 1～21；联合化疗方案主要包括：（1）ICE方案：依托泊苷（100 mg·m^−2^·d^−1^，d 1～3）+卡铂（最大剂量800 mg，d 2）+异环磷酰胺（5 g/m^2^，d 2）；（2）ESHAP方案：依托泊苷（60 mg·m^−2^·d^−1^，d 1～4）+甲泼尼龙（500 mg·m^−2^·d^−1^，d 1～3）+阿糖胞苷（2 g/m^2^，每12 h 1次，d 5）+顺铂（25 mg·m^−2^·d^−1^，d 1～4）；（3）GDP方案：吉西他滨（1 g·m^−2^·d^−1^，d 1、3）+地塞米松（40 mg/d，d 1～4）+顺铂（75 mg/m^2^，d 1）。4例患者桥接方案为泽布替尼+来那度胺±利妥昔单抗：利妥昔单抗375 mg/m^2^，d 0；泽布替尼160 mg每日2次，d 1～21；来那度胺15 mg/d，d 1～14。21 d为1个周期，所有患者接受了1～3个周期泽布替尼为基础的桥接治疗。泽布替尼中位使用时间为2（1～3）个月。所有患者均接受氟达拉滨（25 mg·m^−2^·d^−1^，−5～−3 d）和环磷酰胺（300 mg·m^−2^·d^−1^，−5～−3 d）清除淋巴细胞预处理。第0天开始，通过静脉输注总剂量为3×10^6^/kg的抗CD19 CAR-T细胞（共刺激分子均为4-1BB），1次输注完成。

三、随访与疗效评价

桥接治疗后及CAR-T细胞输注后1、2、3、6、9、12个月行CT检查，CAR-T细胞输注后3、12个月行PET-CT检查评估治疗效果。淋巴瘤疗效评价标准参照参照Lugano淋巴瘤疗效评估标准。通过评估可测量病灶最大垂直径乘积之和（Sum of products of greatest diameters，SPD）缩小情况反应经桥接治疗后肿瘤负荷变化情况。CRS及免疫效应细胞相关中枢神经毒性综合征（Immune effector cell-associated neurotoxicity syndrome，ICANS）的定义和分级根据2022年《CD19 CAR-T治疗B-NHL毒副作用临床管理中国专家共识》[11]进行判定。其他不良反应采用美国不良事件通用术语标准（Common terminology criteria for adverse events，CTCAE）5.0版分级标准进行判定。随访截止日期为2023年6月30日，中位随访24.8（95％ *CI* 17.0～31.6）个月。PFS时间定义为自患者开始口服泽布替尼至疾病进展、复发、死亡或随访截止时间，OS时间定义为自患者开始口服泽布替尼至死亡或末次随访的时间。

四、统计学处理

采用GraphPad Prism 9.0进行数据描述及可视化。连续变量以中位数（范围）进行描述，分类变量以例数（构成比）进行描述；通过Kaplan-Meier法进行生存分析，中位生存时间采用点估算值（95％ *CI*）进行描述。

## 结果

一、基线临床特征

共入组21例伴有高危风险的r/r non-GCB DLBCL患者，大部分为经历过多线治疗失败的患者，伴有高肿瘤负荷大包块，并且基础疾病合并症较多。几乎所有的患者为双表达亚型，且伴有结外累及病灶。患者的具体临床特征见表1。

**表1:** 泽布替尼联合方案桥接CD19 CAR-T细胞治疗21例复发/难治弥漫大B细胞淋巴瘤（DLBCL）患者的临床特征

临床特征	数值
中位年龄［岁, *M*（范围）］	57（38~76）
年龄≥65岁［例（%）］	7（33.3）
男性［例（%）］	15（71.4）
基础疾病［例（%）］	
糖尿病	6（28.5）
心脏病	3（15.8）
慢性阻塞性肺疾病	3（15.8）
IPI评分≥3分［例（%）］	18（85.7）
中位ECOG评分［*M*（范围）］	2（1~3）
Ki-67≥80%［例（%）］	18（85.7）
TP53缺失或突变［阳性例数/总例数（%）］	4/12（33.3）
既往中位治疗线数［*M*（范围）］	3（2~6）
治疗线数≥3线［例（%）］	15（71.4）
肿瘤负荷［例（%）］	
肿块直径≥5 cm	14（66.7）
肿块直径≥10 cm	4（19.0）
DLBCL亚型	
双表达［例（%）］	14（66.7）
C-MYC重排或突变［阳性例数/总例数（%）］	5/16（31.2）
CD5^+^［阳性例数/总例数（%）］	6/21（28.5）
结外受累［例（%）］	19（90.4）
既往治疗方案［例（%）］	
自体干细胞移植	4（19.0）
CAR-T	1（4.8）
放疗	3（14.3）
来那度胺	12（57.1）
西达本胺	2（9.5）
泽布替尼使用时间［月，*M*（范围）］	2（1~3）
泽布替尼联合方案［例（%）］	
来那度胺±利妥昔单抗	4（19.0）
化疗±利妥昔单抗	17（81.0）
CD19 CAR-T细胞共刺激因子	
4-1BB	21（100.0）

注 CAR-T：嵌合抗原受体T细胞；IPI评分：国际预后指数评分；ECOG评分：美国东部肿瘤协作组体能状况评分

二、疗效分析

21例r/r DLBCL患者输注CD19 CAR-T细胞治疗后，11例（52.4％）最佳疗效达CR，6例（28.6％）最佳疗效达部分缓解（PR）。11例获得CR的患者中9例长期无病生存，2例在CD19 CAR-T治疗后1年出现肿瘤复发。在桥接治疗后对所有21例患者进行疗效评估，其中有16例患者达到PR，这部分患者后续经CAR-T治疗后有8例达到了CR，5例达到了PR。13例（61.9％）患者在输注CAR-T细胞前肿瘤负荷明显下降，靶病灶体积（SPD）缩小≥50 ％。21例r/r DLBCL患者的中位PFS时间为12.8（95％*CI* 4.1～NR）个月，中位OS时间未达到；1年PFS率为52.4％（95％*CI* 29.8％～74.3％），1年OS率为80.1％（95％*CI* 58.1％～94.6％）（图1）。

**图1:**
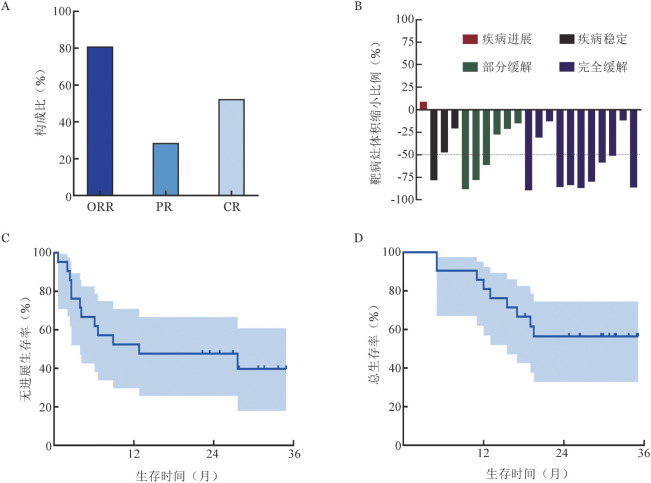
泽布替尼联合方案桥接CD19嵌合抗原受体T细胞（CAR-T）细胞治疗21例复发/难治弥漫大B细胞淋巴瘤患者的疗效分析 A CAR-T 细胞治疗后最佳疗效； B 桥接治疗后靶病灶体积（SPD）缩小比例； C 无进展生存曲线； D 总生存曲线 注 ORR：总反应；PR：部分缓解；CR：完全缓解

三、安全性

18例（85.7％）患者发生了CRS，均为1～2级CRS，无一例发生≥3级CRS。6例（28.6％）出现1级CRS，12例（57.1％）出现2级CRS。所有患者的ICANS发生率较低，仅2例（9.5％）发生轻度ICANS，均为1级ICANS。所有21例患者均发生血液学不良反应，其中4级粒细胞减少11例（52.4％），4级血小板减少7例（33.3％），4级贫血3例（14.3％）；仅5例（23.8％）患者出现轻度肝功能不全，仅1例（4.8％）患者轻度肌酐升高。21例患者中有7例（33.3％）出现凝血功能异常，其中仅1例（4.8％）为3级，其他6例（28.6％）均为1～2级（表2）。

**表2:** 泽布替尼联合方案桥接CD19 CAR-T细胞治疗复发/难治弥漫大B细胞淋巴瘤的不良事件［例（％）］

不良事件	总体	1级	2级	3级	4级
细胞因子释放综合征	18（85.7）	6（28.6）	12（57.1）	0（0）	0（0）
ICANS	2（9.5）	2（9.5）	0（0）	0（0）	0（0）
发热	17（81.0）	6（28.6）	10（47.6）	1（4.8）	0（0）
低血压	8（38.1）	6（28.6）	2（9.5）	0（0）	0（0）
血氧饱和度下降	1（4.8）	1（4.8）	0（0）	0（0）	0（0）
贫血	19（90.5）	11（52.4）	3（14.3）	2（9.5）	3（14.3）
粒细胞减少	21（100.0）	4（19.0）	1（4.8）	5（23.8）	11（52.4）
血小板减少	14（66.7）	4（19.0）	2（9.5）	1（4.8）	7（33.3）
肝功能损害	5（23.8）	5（23.8）	0（0）	0（0）	0（0）
凝血功能异常	7（33.3）	2（9.5）	4（19.0）	1（4.8）	0（0）
肾功能损害	1（4.8）	1（4.8）	0（0）	0（0）	0（0）

注 CAR-T：嵌合抗原受体T细胞；ICANS：免疫效应细胞相关中枢神经毒性综合征

## 讨论

既往的临床试验数据显示，大约7％的患者在等待CAR-T细胞制备过程中死亡[6]，JULIET研究中92％的患者和约80％真实世界的患者接受了桥接治疗[5],[12]，由此可见等待CAR-T治疗的患者迫切需要接受桥接治疗。合适的桥接治疗策略可以控制病情，降低肿瘤负荷，增加患者接受CAR-T细胞治疗的机会。

目前的CAR-T治疗前桥接治疗可分为免疫疗法、化疗联合靶向治疗以及放疗三种，不同桥接治疗桥接后输注CAR-T细胞的疗效并不相同。免疫疗法（如单克隆抗体）能够识别肿瘤细胞上表达的细胞表面抗原，通过招募免疫效应细胞（即巨噬细胞和自然杀伤细胞）介导细胞毒作用[13]。作为桥接治疗方案，CD20单抗、CD79 ADC单抗和抗PD-1抗体在r/r DLBCL患者中具有良好的治疗效果。在一项26个中心的105例B细胞淋巴瘤患者回顾性分析中，28例患者接受维泊妥珠单抗作为桥接治疗并输注CAR-T细胞，6个月的OS率为77.9％，12个月的OS率为58.5％[14]。放疗是一种免疫原性减瘤策略，通过直接破坏肿瘤细胞的DNA来削弱肿瘤细胞分裂和增殖的能力，同时也促进免疫细胞向照射区域的迁移和激活[15]。在一项124例DLBCL患者CAR-T细胞治疗的回顾性研究中，11例桥接放疗患者的中位PFS时间为8.9个月，ORR为100.0％[16]。10例高肿瘤负荷的DLBCL患者在CAR-T治疗前分别桥接化疗或者放疗，其中6例桥接放疗，接受CAR-T治疗后全部患者都获得治疗反应，1例达CR[17]。

化疗是目前r/r DLBCL中最广泛使用的桥接疗法[18]。但是在对接受axi-cel治疗r/r DLBCL患者数据的回顾性分析中，单纯桥接化疗组的PFS（*P*＝0.019）和OS（*P*＝0.001）率明显低于未接受桥接治疗组[19]。另一个大型多中心队列也报道了类似结果，其中54％的高肿瘤负荷DLBCL患者接受了单纯桥接化疗，与未接受桥接化疗的患者相比，输注CAR-T后中位PFS时间分别为3.4个月和7.3个月，中位OS时间为10.3个月和未达到，桥接化疗组的PFS和OS较未桥接组更差[16]。由于需要桥接治疗患者比未桥接患者肿瘤负荷高，疾病进展更快，因此这些回顾性数据存在选择偏倚。

为了克服化疗耐药，越来越多的靶向药物应用于桥接治疗中，包括BTKi、BCL2抑制剂、免疫调节剂、磷脂酰肌醇3-激酶（PI3K）抑制剂和组蛋白去乙酰化酶抑制剂（HDACi）。与传统的化疗药物相比，这些小分子靶向药不受耐药性的影响，并且可以克服某些不良的预后因素。对于non-GCB亚型DLBCL患者而言，BCR信号的激活可促进淋巴瘤细胞的增殖，而BTK在介导BCR的下游信号转导中起着关键性作用[20]。CD79a/b突变及持续激活的BCR信号；而持续激活的BCR通路后续会促进下游4个非受体蛋白酪氨酸激酶家族的持续转录，这4个家族分别是磷脂酶Cγ（PLCγ）、丝裂原活化蛋白激酶（MAPK）、活化B细胞的核因子kappa轻链增强子（NF-кB）和丝氨酸/苏氨酸激酶（AKT）或蛋白激酶B（PKB）[21]。泽布替尼作为新一代BTKi，通过抑制CD79a/b和MYD88高频突变所致活化的BCR和NF-κB通路来降低淋巴瘤细胞的增殖能力，从而杀伤肿瘤细胞[20]。所以本研究采用泽布替尼为基础的联合治疗桥接CAR-T细胞治疗，结果显示泽布替尼为基础的联合桥接方案可以降低肿瘤负荷，顺利桥接到CAR-T治疗。除了对肿瘤本身的抑制作用外，对于患者自身的T细胞而言，在接受BTKi治疗后可以明显改善T细胞的功能缺陷并且增强自身T细胞在体外扩增的能力，并且使与CAR-T细胞增殖能力呈负相关的T细胞上抑制受体PD-1和CD160以及免疫抑制分子CD200的表达降低[9]。BTKi也可以促进CD19 CAR-T细胞的迁移，诱导T细胞分化和肿瘤监视[22]。在肿瘤微环境中，巨噬细胞的极化状态是一个重要因素，M1巨噬细胞表现出促炎抗肿瘤表型，M2巨噬细胞表现出免疫抑制并且抑制T细胞功能[23]。现有研究发现BTKi可以诱导巨噬细胞网从M1分化，并且增加肿瘤相关巨噬细胞的吞噬功能，并且抑制IL-10的产生[24]。此外，髓源性抑制细胞（MDSC）与肿瘤进展密切相关，是肿瘤微环境中重要的免疫抑制因素。现有报道发现BTKi在体外可以抑制MDSC的生成、迁移和分泌TNF-α的功能，同时也可以缓解MDSC介导的CD8^+^ T细胞的抑制状态，增强了抗PD-L1的治疗效果[25]。因此，BTKi可能通过对肿瘤微环境的多方面作用促进CAR-T疗效。ZUMA-1研究中，108例患者接受CAR-T细胞输注，101例可评估病例中，ORR为83％，59例患者获得CR，中位PFS期为5.9个月[26]。JULIET研究中，115例患者接受CAR-T细胞输注后，ORR为53％，中位PFS期为2.9个月[27]。本研究以泽布替尼为基础联合方案桥接CAR-T细胞治疗，ORR为81.0％，11例（52.4％）获得CR，中位PFS期为12.8个月，OS期未达到。本研究中大部分的患者存在CAR-T治疗预后不良的重要因素，例如高肿瘤负荷、Ⅲ/Ⅳ期、ECOG≥2分等，但获得了与ZUMA-1及JULIET研究相似的有效率和更长的PFS时间，这可能与本研究中应用以泽布替尼为基础的桥接方案有关。

本研究没有动物及细胞学实验来证实泽布替尼对于CAR-T细胞功能和活性的影响是本文的不足之处。其次，本研究为单臂临床研究，未进行随机对照研究，样本量较小，存在一定的局限性。

综上所述，泽布替尼为基础的联合治疗作为CAR-T前的桥接方案，减小了患者的肿瘤负荷，阻碍了肿瘤的生长，为部分难以过渡到CAR-T细胞治疗的患者提供了可能的治疗方法。此外，本研究显示接受泽布替尼为基础的联合方案桥接CAR-T治疗r/r non-GCB DLBCL患者较以往的CAR-T临床研究具有更长的PFS时间，因此以泽布替尼为基础的联合治疗作为桥接方案可提高CAR-T治疗r/r DLBCL的疗效。
